# Supplementary material for: Content-rich biological network constructed by mining PubMed abstracts
Source: BMC Bioinformatics. 2004 Oct 8;5:147. doi: 10.1186/1471-2105-5-147 (PMC528731; doi:10.1186/1471-2105-5-147)
Supplement: Additional File 5 — The original Chilibot query results of the term "long-term potentiation (LTP)" and 22 other terms, limiting the latest references analyzed to the years 1990, 1995, 2000, and 2004. [file 1471-2105-5-147-S5.bz2 › chilibotAdditionalFile5/ltp2000/html/ERK_PKC.html]

 


 **ERK** and **PKC** 
  
Found 217 abstracts in PubMed,  **30 abstracts were retrieved and analyzed**.  


---

 Search Google  |
 PDF files only 
|  EDU domain only 

---

**Interactive relationship** (e.g. stimulation, inhibition, etc)

- We found that a peptide specific for  **PKC**  zeta partially blocked activation of both MEK and  **ERK**  by LPS.  Ref: 11035106 J Immunol, 2000
- While phorbol ester induced extracellular signal regulated kinase activation required protein kinase C activity, preincubation of H69 cells with the  **PKC**  inhibitor GF109203X had no effect on galanin dependent  **ERK**  activity.  Ref: 10980593 Oncogene, 2000
- Extracellular signal regulated kinase  **ERK**  activation in CTL stimulated with soluble cross linked anti CD3 is completely inhibited by the  **PKC**  inhibitor bisindolylmaleimide BIM.  Ref: 11120810 J Immunol, 2000
- Unlike many effects of HG, augmentation of  **ERK**  activity by HG was not dependent on protein kinase C as indicated by downregulation of  **PKC**  with 24 h phorbol ester or inhibition with bisindolylmaleimide IV.  Ref: 10997919 Am J Physiol Renal Physiol, 2000
- the overexpression of  **PKC**  delta induces  **ERK**  1 2 activation.  Ref: 11031252 J Biol Chem, 2001
- By using different  **PKC**  inhibitors and a dominant negative  **PKC**  delta, it was found that  **ERK**  activation was  **PKC**  delta dependent.  Ref: 11031252 J Biol Chem, 2001
- We conclude that phosphorylation of the M 3 muscarinic receptor on sites in the third intracellular loop by casein kinase 1alpha contributes to the mechanism of receptor activation of  **ERK**  1 2 by working in concert with the diacylglycerol  **PKC**  arm of the phospholipase C signaling pathway.  Ref: 11083874 J Biol Chem, 2001
- We show that extracellular signal regulated kinase  **ERK**  activation is fully dependent on protein kinase C  **PKC** , but only partially dependent on Src, dynamin, and Ras.  Ref: 11083862 J Biol Chem, 2001
- Further analysis of the signaling events induced by IgG opsonized sheep red blood cells revealed that whereas tyrosine phosphorylation of Syk was normal, phosphorylation of  **ERK**  1 2 p42 44 was impaired in DN  **PKC**  alpha overexpressing macrophages.  Ref: 11027499 Biochem Biophys Res Commun, 2000
- Furthermore, both of the experimental manipulations serum deprivation and  **PKC**  inhibition synergically, but to different extents, suppressed the extracellular signal regulated kinase  **ERK**  pathway.  Ref: 11057029 Folia Neuropathol, 2000
- We conclude, therefore, that apoptosis of N2a cells triggered by  **PKC**  inhibition, as well as its abrogation by serum, is totally independent of concomitant modulations of  **ERK**  activity also evoked by the above treatments.  Ref: 11057029 Folia Neuropathol, 2000
- Both  **ERK**  and cPLA 2 phosphorylation in response to thapsigargin were inhibited by PD 98059, a specific inhibitor of MAP kinase kinase of the  **ERK**  group MEK, and EGTA. cPLA 2 phosphorylation was not affected by Ro 31 8220 an inhibitor of all  **PKC**  isoforms or LY 379196 a PKCbeta selective inhibitor, while both of them indeed attenuated  **ERK**  activation.  Ref: 10996851 J Cell Biochem, 2000
- ERK1 2 activation by IL 1beta was sensitive to inhibition by the  **PKC**  inhibitor bisindolylmaleimide suggesting that  **ERK**  phosphorylation is a downstream signal of  **PKC**  activation.  Ref: 10960082 Br J Pharmacol, 2000

**Parallel relationship** (e.g. studied together, co-existance, homology, etc.)

- Alternatively, the  **PKC**  MEK1  **ERK**  pathway in the early stage plays a significant role in up regulation of the MSR gene.  Ref: 10969071 J Biol Chem, 2000
- In the present work the differential activation of  **PKC**  isoforms and of signal transduction pathways, including nerve growth factor receptor trkA, phospholipase Cgamma 1 PLCgamma 1, and extracellular regulated kinases 1 and 2  **ERK**  1 2 by TeTx in a synaptosome enriched P 2 fraction from rat brain is reported.  Ref: 10996319 FEBS Lett, 2000
- We conclude that UTI markedly suppresses the cell motility possibly through negative regulation of  **PKC**  and MEK  **ERK**  c Jun dependent mechanisms,.  Ref: 11050091 J Biol Chem, 2001
- These data suggest that VEGF can increase CTGF gene expression in bovine retinal capillary cells via KDR or Flt receptors and the activation of PI3 kinase Akt pathway independently of  **PKC**  or Ras  **ERK**  pathway, possibly inducing the fibrosis observed in retinal neovascular diseases.  Ref: 11018037 J Biol Chem, 2000
- The purpose of this study was to determine whether mitogen activated protein kinase MAPK, extracellular signal regulated kinase  **ERK**  1 and ERK2, or protein kinase C  **PKC**  activation is required for H 2 O 2 induced contraction.  Ref: 10993784 Am J Physiol Heart Circ Physiol, 2000
- Surprisingly, MAPK activation in response to immobilized anti CD3 is strongly inhibited at all time points by the diacylglycerol DAG binding domain inhibitor calphostin C implicating the contribution of a DAG dependent but  **PKC**  independent pathway in the activation of  **ERK**  in CTL clones.  Ref: 11120810 J Immunol, 2000
- Using specific kinase inhibitors and kinetic analyses, we provide evidence for two distinct components in the activation of  **Erk**  by Gi and Gq protein coupled receptors in MC3T3 E1 cells including an Src like kinase dependent pathway and a protein kinase C  **PKC**  dependent mechanism.  Ref: 10976990 J Bone Miner Res, 2000
- In A549 cells,  **PKC**  activator PMA cannot increase either the basal or thapsigargin induced 3 H AA release, while it can induce the phosphorylation of  **ERK**  and cPLA 2.  Ref: 10996851 J Cell Biochem, 2000
- **ERK**  44 42 activation by CPO is insensitive to the protein kinase A inhibitor H 89, but is completely abolished by the phosphatidylinositol 3 kinase P13 K inhibitor wortmannin, the protein kinase C  **PKC**  inhibitor GF 109203X, and the mitogen activated extracellular signal regulated protein kinase kinase MEK inhibitor PD 098059.  Ref: 11083088 J Biochem Mol Toxicol, 2000
- Moreover, deficiency in fyn dependent PLC gamma1 catalytic activity may contribute to reduced  **PKC**  alpha dependent  **ERK**  activation.  Ref: 11093170 Eur J Immunol, 2000
- Inhibitors specific for mitogen activated protein  **ERK**  kinase 1 2 MEK1 2, protein kinase C  **PKC** , and Ras and co transfections of wild type and mutant Raf were used to elucidate the signaling cascades involved.  Ref: 11069776 J Cell Sci, 2000
- ...  **PKC**  and  **ERK**  pathways made only minimal contributions.  Ref: 11018037 J Biol Chem, 2000
- Taken as a whole, these studies suggest that LPS activates  **ERK**  kinase, in part, through activation of an atypical  **PKC**  isoform,  **PKC**  zeta.  Ref: 11035106 J Immunol, 2000
- ... the blockade of  **PKC** , PKA, and  **ERK**  had little effect.  Ref: 11006016 Cell Immunol, 2000
- In this study, we evaluated the role of the atypical protein kinase C  **PKC**  isoform,  **PKC**  zeta, in LPS induced activation of the  **ERK**  kinase pathway.  Ref: 11035106 J Immunol, 2000
- In addition, protein kinase C dependent signaling pathways contribute to CCK mediated MAP kinase signaling as shown by inhibition of CCK 8 induced  **ERK**  activation by the  **PKC**  inhibitor bisindolylmaleimide.  Ref: 11030434 Biol Chem, 2000
- Activation of signal transduction pathways involving trkA, PLCgamma 1,  **PKC**  isoforms and  **ERK**  1 2 by tetanus toxin.  Ref: 10996319 FEBS Lett, 2000
- The results in this paper indicate that LPS induced NF kappa B binding activity was independent of  **PKC** , PKA,  **ERK** , and p38 MAPK, but was regulated by proteasome.  Ref: 11006016 Cell Immunol, 2000
- Phosphorylation consensus sites for various kinases exist within the sequence of the potassium channel subunit Kv4.2, including consensus sites for extracellular signal regulated kinase mitogen activated protein kinase  **ERK**  MAPK, protein kinase A PKA, protein kinase C  **PKC** , and calcium calmodulin dependent kinase II CaMKII, and kinase assays have shown that particular amino acids of the consensus sites are bonafide phosphorylation sites in vitro.  Ref: 11040264 Learn MemLearn Mem, 2000
- After mice were infected daily with 30 third stage larvae of A. cantonensis by oral adminstration for 6 weeks, no significant differences  **PKC**  alpha, MEK 1,  **ERK**  2, JNK, and p38 protein expression were found between the control and infected mice.  Ref: 10964648 Exp Parasitol, 2000
- Kinase activity assays showed that LPS activates  **PKC**  zeta, mitogen activated protein  **ERK**  kinase MEK, the upstream activator of  **ERK** , and  **ERK** .  Ref: 11035106 J Immunol, 2000
- Therefore, CPO activates the  **ERK**  44 42 signaling cascade in CHOK1 cells via a pathway dependent on P13 K,  **PKC** , and MEK but not requiring PKA or the human M2 muscarinic receptor.  Ref: 11083088 J Biochem Mol Toxicol, 2000
